# Muscle strength adaptation between high-load resistance training versus low-load blood flow restriction training with different cuff pressure characteristics: a systematic review and meta-analysis

**DOI:** 10.3389/fphys.2023.1244292

**Published:** 2023-08-25

**Authors:** Hualong Chang, Jing Yan, Guiwei Lu, Biao Chen, Jianli Zhang

**Affiliations:** ^1^ College of Physical Education and Health Sciences, Zhejiang Normal University, Jinhua, China; ^2^ College of Education, Anyang Normal University, Anyang, China; ^3^ Exercise and Metabolism Research Center, College of Physical Education and Health Sciences, Zhejiang Normal University, Jinhua, China

**Keywords:** blood flow restriction, muscle strength, occlusion pressure prescriptions, cuff inflation patterns, adult

## Abstract

**Purpose:** In this systematic review and meta-analysis, blood flow restriction (BFR) with low-load resistance training (BFR-RT) was compared with high-load resistance training (HL-RT) on muscle strength in healthy adults. The characteristics of cuff pressure suitable for muscle strength gain were also investigated by analyzing the effects of applying different occlusion pressure prescriptions and cuff inflation patterns on muscle strength gain.

**Methods:** Literature search was conducted using PubMed, Ovid Medline, ProQuest, Cochrane Library, Embase, and Scopus databases to identify literature published until May 2023. Studies reporting the effects of BFR-RT interventions on muscle strength gain were compared with those of HL-RT. The risk of bias in the included trials was assessed using the Cochrane tool, followed by a meta-analysis to calculate the combined effect. Subgroup analysis was performed to explore the beneficial variables.

**Results:** Nineteen articles (42 outcomes), with a total of 458 healthy adults, were included in the meta-analysis. The combined effect showed higher muscle strength gain with HL-RT than with BFR-RT (*p* = 0.03, SMD = −0.16, 95% CI: −0.30 to −0.01). The results of the subgroup analysis showed that the BFR-RT applied with incremental and individualized pressure achieved muscle strength gain similar to the HL-RT (*p* = 0.8, SMD = −0.05, 95% CI: −0.44 to 0.34; *p* = 0.68, SMD = −0.04, 95% CI: −0.23 to 0.15), but muscle strength gain obtained via BFR-RT applied with absolute pressure was lower than that of HL-RT (*p* < 0.05, SMD = −0.45, 95% CI: −0.71 to −0.19). Furthermore, muscle strength gain obtained by BFR-RT applied with intermittent pressure was similar to that obtained by HL-RT (*p* = 0.88, SMD = −0.02, 95% CI: −0.27 to 0.23), but muscle strength gain for BFR-RT applied with continuous pressure showed a less prominent increase than that for HL-RT (*p* < 0.05, SMD = −0.3, 95% CI: −0.48 to −0.11).

**Conclusion:** In general, HL-RT produces superior muscle strength gains than BFR-RT. However, the application of individualized, incremental, and intermittent pressure exercise protocols in BFR-RT elicits comparable muscle strength gains to HL-RT. Our findings indicate that cuff pressure characteristics play a significant role in establishing a BFR-RT intervention program for enhancing muscle strength in healthy adults.

**Clinical Trial Registration:** https://www.crd.york.ac.uk/PROSPERO/#recordDetails; Identifier: PROSPERO (CRD42022364934).

## 1 Introduction

Muscle strength is important for normal survival and daily living. It is also considered an important predictor of cardiometabolic risk and is associated with morbidity in adults and older adults (Silventoinen et al., 2009). Studies have identified low grip strength as a potential risk factor for skeletal sarcopenia, functional limitation, and disability (Rantanen et al., 1999; Manini and Clark, 2012); it is also considered a valuable indicator of frailty in older adults (Takahashi et al., 2017). In addition, a meta-analysis study of data from approximately 20,000 men and women associated high levels of upper and lower limb muscle strength with a reduced risk of mortality in adults (García-Hermoso et al., 2018). Another follow-up study over a period of 33 years showed a negative association between muscle strength and all-cause mortality (Stenholm et al., 2014). This correlation was also observed in people with specific diseases, including cardiovascular disease, peripheral arterial disease, cancer, renal failure, chronic occlusive lung disease, and rheumatoid arthritis, and among critically ill patients (Jochem et al., 2019). Therefore, muscle strength can be regarded as a potential predictor of morbidity and mortality risk in the general population (Leon et al., 2005; Peterson and Krishnan, 2015), and it plays a key role in protecting human health.

The American College of Sports Medicine previously suggested that the use of high-load resistance training [(HL-RT, ≥70% 1RM)] is an effective means of promoting muscle strength gain (Garber et al., 2011). However, this training method has drawbacks, with HL-RT exercise increasing the likelihood of sports-related injuries. Furthermore, for patients undergoing rehabilitation, HL-RT exercise is not recommended in the early stages of recovery, as it may increase the risk of reinjury (Hughes et al., 2019). In elderly individuals, the presence of comorbidities, such as coronary heart disease, diabetes, or musculoskeletal injuries, may further complicate the use of HL-RT (Papa et al., 2017). However, low-load exhaustive exercise, while also effective in improving muscle strength, may cause subjective discomfort in older adults and rehabilitating patients. Moreover, prolonged recovery time from fatigue may lead to red blood cell abnormalities, increasing the likelihood of tissue damage or the exacerbation of existing injuries (Brun et al., 1994). Emerging blood flow restriction (BFR) training with low-load resistance training (BFR-RT) techniques can circumvent these problems.

BFR training, also known as KAATSU training or vascular occlusion training, refers to the external compression of the limb during exercise by using a special compression device that causes venous occlusion and partial arterial occlusion to improve the training effect (Sato, 2005; Loenneke et al., 2014). BFR-RT produces a local metabolic crisis via physical compression, which induces the brain to secrete growth hormones to promote anabolism, improve muscle strength, and accelerate tissue repair (Fry et al., 2010). Common practice dictates that more muscle strength gain can be obtained with BFR-RT than with low-load resistance training. However, controversy arises when BFR-RT is compared with HL-RT, which is known as the gold standard for increasing muscle strength. For example, some studies have shown that BFR-RT training can produce muscle strength gain similar to HL-RT (Libardi et al., 2015; Centner et al., 2022; Chang et al., 2022), whereas other studies have shown that BFR-RT training leads to less muscle strength gain than HL-RT (Lixandrao et al., 2018; Sieljacks et al., 2019).

It is necessary to discuss these controversies based on cuff pressure characteristics. A meta-analysis explored the effect of using different occlusion pressure prescriptions (individualized and non-individualized pressures) on muscle strength gain during BFR-RT exercise, results indicated that the BFR-RT group demonstrated lower muscle strength gain than the HL-RT group, even after controlling for the abovementioned factors (Lixandrao et al., 2018). Interestingly, however, Individualized pressure appears to be widely accepted owing to its logical basis, but it does not justify the choice of individualized pressure when setting up a BFR-RT exercise program, and more theoretical support needs to be found for the use of individualized pressure for BFR-RT (Clarkson et al., 2020). Another related meta-analysis (Sinclair et al., 2022) described that the cuff inflation pattern (continuous and intermittent pressure) was not a variable affecting muscle strength gain, but the results of the analysis were limited to two articles, entailed limited guidance value in terms of enhancing muscle strength with BFR-RT interventions that use different cuff inflation patterns. In addition. To our knowledge, no meta-analysis has yet considered the effects of BFR-RT occlusion pressure prescription, cuff inflation mode, and participant characteristics on muscle strength adaptation simultaneously.

Therefore, the objectives of our study were threefold. Firstly, we aimed to examine the adaptation of muscle strength in individuals undergoing BFR-RT compared to those undergoing HL-RT. Secondly, we aimed to analyze the impact of occlusion pressure prescription and cuff inflation pattern on the adaptation of muscle strength. Lastly, we sought to investigate the cuff pressure characteristics that are suitable for promoting muscle strength adaptation in diverse participants.

## 2 Methods

The systematic review was performed according to the latest guidelines of Preferred Reporting Items for Systematic Reviews and Meta-Analyses (PRISMA) (Page et al., 2021). This study was also registered with PROSPERO (CRD42022364934).

### 2.1 Search strategy

A systematic literature search was conducted by two researchers (HC and GL) to identify relevant studies. The following electronic databases were searched from database creation to 31 December 2022: PubMed, Ovid Medline, ProQuest, Cochrane Library, Embase, and Scopus. The following specific keywords were used to search the articles: “blood flow restriction training” OR “Kaatsu training” OR “occlusion training” OR “vascular occlusion” OR “KAATSU” OR “blood flow occlusion” AND “muscle strength” OR “muscle force” OR “dynamic” OR “isokinetic” OR “muscle power” AND “randomized controlled trial” OR “RCT.” We conducted an additional search on 20 May 2023, to identify potential studies published since the last search. The detailed search strategy implemented in the PubMed database is provided in Supplementary Table S1. In addition, we manually searched the lists of references to obtain other suitable articles.

### 2.2 Inclusion and exclusion criteria

The selection criteria were established based on Participants, Interventions, Control, Outcome, and Study design (PICOS).

**FIGURE 1:**
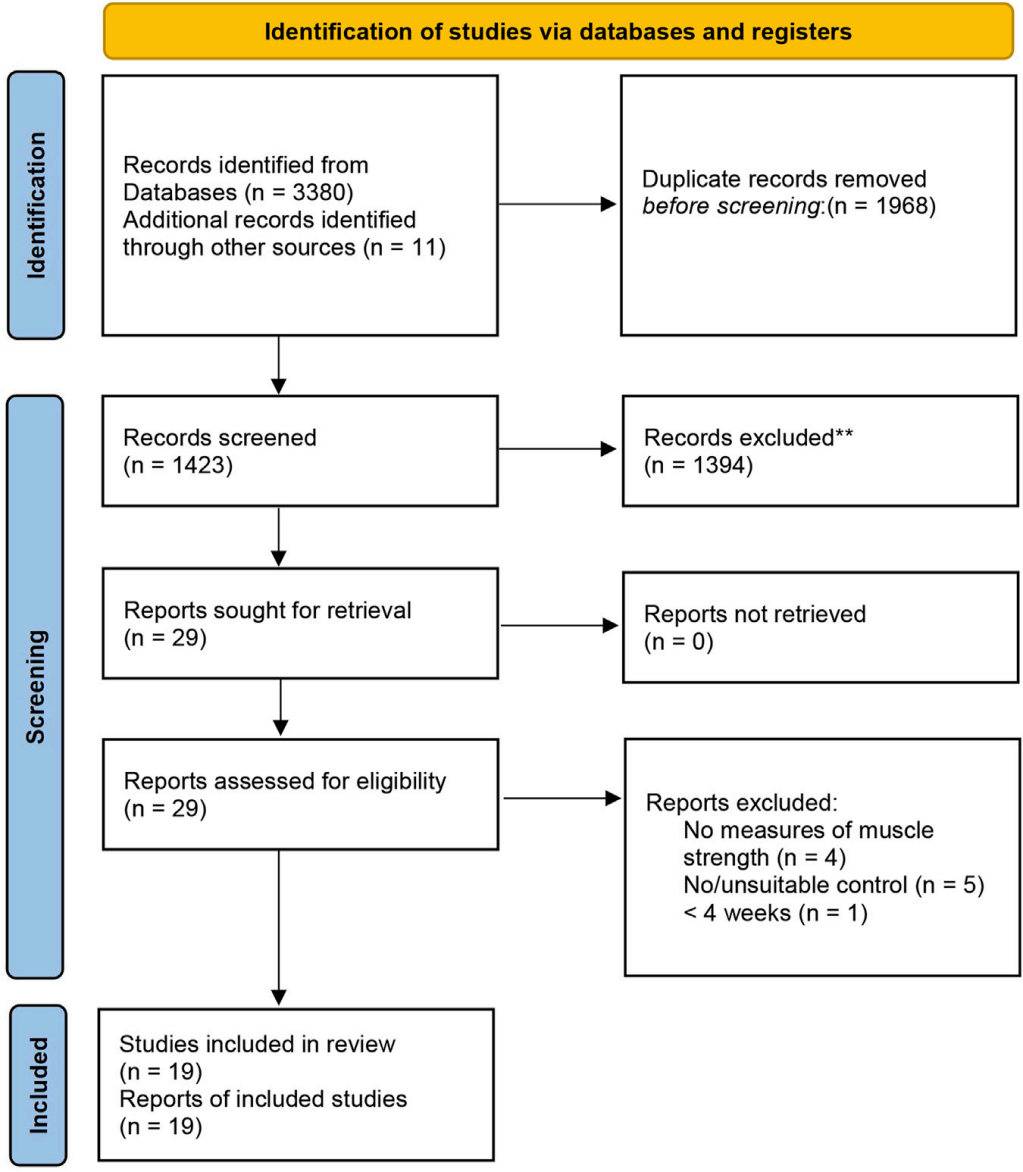
Flowchart of the study selection according to the latest Preferred Reporting Items for Systematic Review and Meta-Analysis (PRISMA).

Before inclusion, the titles and abstracts of the retrieved articles were screened for relevance. Subsequently, the full texts of the articles were obtained and reviewed based on the inclusion criteria. To determine the articles to be included in this study, we adopted the following inclusion criteria: 1) participants: healthy adults (age ≥18 years); 2) control group [resistance training without vascular occlusion (HL-RT) at ≥70% 1RM] and intervention group [resistance training with vascular occlusion (BFR-RT) at <50% 1RM)]; 3) outcome: before the start of the experiment and at the end of the experiment to assess muscle strength (i.e., dynamic, isometric, or isokinetic test); and 4) study design: the studies should be RCTs with parallel-groups. We excluded studies based on the following criteria: 1) studies conducted with animals as subjects; 2) trials involving drug supplements affecting muscle strength; 3) the experimental group and the control group were the left and right sides of the experimental subject; 4) non-original research articles (experimental protocols, meta-analyses, and systematic reviews); and 5) articles not published in English. Relevant articles were reviewed and assessed independently by two authors (HC and GL) according to the inclusion/exclusion criteria. First, the titles and abstracts of the identified articles were screened for relevance. Second, the full text of specific articles was obtained, and the full text of each article was fully assessed against the inclusion criteria. Any discrepancies were resolved by discussion with another author (JZ) to reach a final consensus. The detailed study selection process according to the PRISMA guidelines is shown in Figure 1.

### 2.3 Outcome measures and data extraction

The study characteristics were extracted independently by two authors (CH and JY) by using an Excel spreadsheet. The following characteristics were extracted from each article: 1) surname of the first author; 2) characteristics of participants (number and gender); 3) exercise/intervention characteristics (exercise load, frequency, and duration); 4) muscle strength tests (i.e., dynamic, isometric, and isokinetic); and 5) proportion of muscle strength gain and comparison between experimental and control groups. When the article provided data in graph format, the data were extracted via the Web Plot Digitizer. The percentage change in muscle strength [((Mean_post_ − Mean_pre_)/Mean_pre_) × 100] was calculated for each study. In the case of multiple assessment methods, the minimum and maximum mean values for each method were reported (Table 1). Notably, in the assessment of post-intervention muscle strength across multiple periods, the analyses were based on the last available time point. In case of any disagreement between the two authors regarding the included features, a third reviewer (JZ) was consulted. Finally, the extracted features were cross-checked and analyzed in depth by the other authors. To obtain missing or additional data, the corresponding author was contacted.

**TABLE 1:** Characteristics of the included studies.

Study	Participant characteristics		Intervention	Training protocol	Duration (weeks, sessions)	Strength measurement	Outcomes	Intergroup comparison
	Age	Sample Size(M/F)						
Bemben et al. (2022)	21.3 ± 2.5	BFR-RT, 12/0	20% 1RM	30.3 × 15, 60s	6 (18)	Dynamic knee extension Dynamic knee flexion	BFR-RT: 19%	→
	20.9 ± 2.9	HL-RT, 12/0	70% 1RM	3 × 10, 60s			HL-RT: 25%–32%	
Brandner et al. (2019)	23.0 ± 3.0	BFR-RT, 8/3	20% 1RM	30.3 × 15; or 3 × 15, 60s	8 (20)	Dynamic knee extension Dynamic back squat Dynamic calf raises Dynamic bench press Dynamic seated row Dynamic biceps curl	BFR-RT: 6%–21%	↓
	23.0 ± 3.0	HL-RT, 7/4	70% 1RM	3–4×8–10, 60s			HL-RT: 13%–25%	
Centner et al. (2019)	27.1 ± 4.7	BFR-RT, 11/0	20%–35% 1RM	30.3 × 15, 60s	14 (42)	Isometric plantar flexion	BFR-RT: 10%	→
	26.1 ± 4.2	HL-RT, 14/0	70%–85% 1RM	3×6–12, 60s			HL-RT: 14%	
Centner et al. (2022)	28.4 ± 4.9	BFR-RT, 14/0	20%–35% 1RM	30.3 × 15, 60s	14 (42)	Dynamic leg press Dynamic knee extension	BFR-RT:34%–51%	→
	27.6 ± 4.3	HL-RT, 15/0	70%–85% 1RM	3×6–12, 60s			HL-RT:37%–38%	
Centner et al. (2023)	28.4 ± 4.9	BFR-RT, 14/0	20%–35% 1RM	30.3 × 15, 60s	14 (42)	Dynamic plantar flexors	BFR-RT: 43.6%	→
	27.6 ± 4.3	HL-RT, 15/0	70%–85% 1RM	3×6–12, 60s			HL-RT: 43.5%	
Clark et al. (2011)	23.7 ± 1.4	BFR-RT, 8/1	30% 1RM	3×failure, 90s	4 (12)	Isometric knee extension	BFR-RT: 8%	→
	24.3 ± 1.8	HL-RT, 6/1	80% 1RM	3×failure, 90s			HL-RT: 13%	
Horiuchi et al. (2023)	22.0 ± 2.0	BFR-RT, 12/0	30% 1RM	4 × 20, 30s	4 (16)	Dynamic knee extension Dynamic leg press	BFR-RT: 12%–14%	→
	22.0 ± 2.0	HL-RT, 12/0	75% 1RM	3 × 10, 120s			HL-RT: 14%–15%	
Karabulut et al. (2010)	55.9 ± 1.0	BFR-RT, 13/0	20% 1RM	30.2 × 15, 60s	6 (18)	Dynamic leg press Dynamic leg extension	BFR-RT: 19%	↓
	57.5 ± 0.8	HL-RT, 13/0	80% 1RM	3 × 8, 60s			HL-RT: 20%–31%	
Laswati et al. (2018)	33.0 ± 3.1	BFR-RT, 6/0	30% 1RM	30.3 × 15, 30s	5 (10)	Isokinetic elbow flexion	BFR-RT: 40%	↑
	33.33 ± 3.14	HL-RT, 6/0	70% 1RM	3 × 12, 120s			HL-RT: 36%	
Laurentino et al. (2012)	20.0 ± 4.5	BFR-RT, 10/0	20% 1RM	3–4×15, 60s	8 (16)	Dynamic knee extension	BFR-RT: 40%	→
	23.6 ± 6	HL-RT, 9/0	80% 1RM	3–4×8, 60s			HL-RT: 36%	
Letieri et al. (2018)	68.7 ± 4.8	BFR-RT, 0/22	20%–30% 1RM	3–4×15, 30s	16 (48)	Isokinetic knee extension	BFR-RT: 16%–28%	→
	66.75 ± 4.43	HL-RT, 0/10	70%–80% 1RM	3–4×6–8, 60s			HL-RT:28%–30%	
Libardi et al. (2015)	64.0 ± 4.0	BFR-RT, N = 10	20%–30% 1RM	30.3 × 15, 60s	12 (24)	Dynamic leg press	BFR-RT: 21%	→
	65.0 ± 3.7	HL-RT, N = 8	70%–80% 1RM	4 × 10, 60s			HL-RT: 37%	
Lixandrão et al. (2015)	27.9 ± 8.3	BFR-RT,43/0	20%–40% 1RM	2–3×15, 60s	12 (24)	Dynamic knee extension	BFR-RT: 10%–13%	↓
	29.2 ± 9.9	HL-RT, 9/0	80% 1RM	2–3×10, 60s			HL-RT: 22%	
Martín-Hernández et al. (2013)	20.7 ± 1.6	BFR-RT, 20/0	20% 1RM	30.3 × 15, 60s	5 (10)	Dynamic knee extension Isokinetic knee extension	BFR-RT: 6%–7%, 2%–6%	↓
	20.7 ± 2.3	HL-RT, 11/0	85% 1RM	3 × 8, 60s			HL-RT: 18%, 7%–8%	
Mendonca et al. (2021)	22.3 ± 2.9	BFR-RT, 8/7	20% 1RM	30.3 × 15, 30s	4 (20)	Isometric plantar-flexion	BFR-RT: 16%	→
	21.9 ± 3.3	HL-RT, 8/7	75% 1RM	4 × 10, 60s			HL-RT: 18%	
Ozaki et al. (2013)	23.0 ± 0	BFR-RT, 10/0	30% 1RM	30.3 × 15, 30s	6 (18)	Dynamic bench press	BFR-RT: 9%	→
	24.0 ± 1.0	HL-RT, 9/0	75% 1RM	3 × 10, 120–180s			HL-RT: 18%	
Thiebaud et al. (2013)	59.0 ± 2.0	BFR-RT, 0/6	10%–30% 1RM	30.2 × 15, 30s	8 (24)	Dynamic chest press Dynamic seated row Dynamic shoulder press	BFR-RT: 5%–10%	→
	62.0 ± 2.0	HL-RT, 0/8	70%–90% 1RM	3 × 10, 60–120s			HL-RT: 5%–18%	
Vechin et al. (2015)	65.0 ± 2.0	BFR-RT, N = 8	20%–30% 1RM	30.3 × 15, 60s	12 (24)	Dynamic leg press	BFR-RT: 17%	↓
	68.7 ± 15.3	HL-RT, N = 8	70%–80% 1RM	4 × 10, 60s			HL-RT: 54%	
Yasuda et al. (2011)	23.4 ± 1.3	BFR-RT, 10/0	30% 1RM	30.3 × 15, 30s	6 (18)	Dynamic bench press Isometric elbow extension	BFR-RT: 9%, 0	↓
	25.3 ± 2.9	HL-RT, 10/0	75% 1RM	3 × 10, 120–180s			HL-RT: 20%, 11%	

M, male; F, female; BFR-RT, blood flow restriction combined with low load resistance training; HL-RT, high load resistance training; →, No significant between-group difference; ↓, Trend toward greater muscle strength gains for HL-RT, ↑, Trend toward greater muscle strength gains for BFR-RT.

### 2.4 Quality assessment for the included trials

The Cochrane risk of bias tool (Higgins et al., 2011) was used in this study. The tool includes the following items: random sequence generation/assignment concealment (selection bias), subject/person blinding (performance bias), outcome assessment blinding (detection bias), incomplete outcome data (attrition bias), selective reporting (reporting bias), and other biases. The quality of each domain was rated as “low risk,” “high risk,” or “unclear” and was indicated by green (+), red (−), and yellow (?) colors and symbols. The quality of the trial was assessed by two reviewers (HC and JY). In case of disagreement, consensus was reached through discussion. Persistent differences were resolved by discussion with a third reviewer (JZ) to reach a consensus.

### 2.5 Statistical analysis

Review Manager (RevMan 5.4., Copenhagen, Denmark) was used to statistically analyze the effect of BFR-RT intervention on muscle strength gain in healthy adults. Due to significant differences between the measurement tools and units of each outcome index, the standardized mean differences (SMDs) and 95% confidence intervals (95% CIs) were calculated using the random effect model to summarize the outcome indicators. The I^2^ statistic was used to determine the heterogeneity. For each comparison, the pooled effect size (ES) calculated, and the alpha level was set at *p* < 0.05. Data are reported as mean ± standard deviation. The I^2^ was labeled as having low heterogeneity when the value was less than 25%, moderate heterogeneity when the value was between 25% and 75%, and high heterogeneity when the value was greater than 75%.

This research first compared the effects of the BFR-RT and HL-RT groups in terms of muscle strength gain. A subgroup analysis of cuff pressure characteristics (occlusion pressure prescription and cuff inflatable pattern) was also conducted to examine the effect of different occlusion pressure prescriptions (individualized, incremental, and absolute pressures) and cuff inflatable patterns (continuous and intermittent pressures) on muscle strength gain. The inclusion criteria for subgroups are shown in Supplementary Table S2. On this basis, we further analyzed the effect of participants’ age and gender on subgroup outcomes. In addition, also used Origin (Origin 2021; Massachusetts, United States) to draw 3D images to investigate the combined effect on muscle strength gain.

The sensitivity analysis was performed via a meta-analysis after removing each study to determine if any of the studies were biased against the combined results. A study was considered to bias the pooled results when the estimate after the removal of a study exceeded the 95% CI for the joint effect. An Egger test was performed using Stata version 12 (StataCorp., College Station, TX, United States), and a funnel plot was constructed to check for potential bias in the included RCTs (Supplementary Figure S1).

## 3 Results

### 3.1 Search results and selection of studies

A total of 3391 articles were identified in the systematic search. Among them, 3380 articles were from electronic databases (PubMed, Ovid Medline, ProQuest, Cochrane Library, Embase, and Scopus databases), and 11 articles were from other sources (i.e., manual searches of the included reference lists of included studies and related reviews). After removing duplicates (1968 articles), 1423 articles were obtained for subsequent evaluation. A careful review of the titles and abstracts of the 1423 articles led to the exclusion of an additional 1394 articles. Finally, the full texts of the remaining articles were thoroughly checked. Nineteen articles met the requirements of the qualitative analysis. Figure 1 shows the process flowchart.

### 3.2 Summary of the included studies

#### 3.2.1 Study characteristics and participants

19 articles were selected for analysis based on the inclusion and exclusion criteria, all of which were published between 2010 and 2023. Among them, 19 articles had subjects with no training experience or no recent (≥2 months) systematic resistance training. We included a total of 458 subjects, 257 in the HL-RT group and 201 in the BFR-RT group. Of these, 2 articles had female subjects, totaling 46 and they’re all elderly, with a mean age of 64.54 years; 12 articles had male subjects, totaling 310, ranging in age from 18 to 64 years, with a mean age of 27.82 years; and 3 articles that did not differentiate between the genders of the subjects included 45 males and 23 females, all of whom were young adults, with an average age of 23.02 years; 2 articles failed to provide information regarding the participants’ gender, encompassing a total of 34 individuals who were all elderly adults, with a mean age of 64.37 years.

#### 3.2.2 Intervention characteristics

The exercise load in all articles was between 20% and 40% 1RM for the BFR-RT group and between 70% and 90% 1RM for the HL-RT group. 6 articles had a training frequency of 2 days/week, 11 articles had a training frequency of 3 days/week, 1 article had a training frequency of 4 days/week, and 1 article had a training frequency of 5 days/week. The overall training period was 4–16 weeks 12 articles showed that BFR-RT produced comparable muscle strength gain to HL-RT (Clark et al., 2011; Laurentino et al., 2012; Ozaki et al., 2013; Thiebaud et al., 2013; Libardi et al., 2015; Letieri et al., 2018; Centner et al., 2019; Mendonca et al., 2021; Bemben et al., 2022; Centner et al., 2022; Centner et al., 2023; Horiuchi et al., 2023); 6 articles showed that BFR-RT produced lower muscle strength gain than HL-RT (Karabulut et al., 2010; Yasuda et al., 2011; Martín-Hernández et al., 2013; Lixandrão et al., 2015; Vechin et al., 2015; Brandner et al., 2019), and 1 article showed that BFR-RT could lead to greater muscle strength gain than HL-RT (Laswati et al., 2018). Of all the articles, occlusion pressure prescription applied individualized pressure in 10 articles, incremental pressure in 5 articles, and absolute pressure in 5 articles; inflation mode applied intermittent pressure in 7 articles and continuous pressure in 13 articles. Table 1 and Supplementary Table S5 show the population characteristics and exercise characteristics.

### 3.3 Summary of risk of bias

**FIGURE 2:**
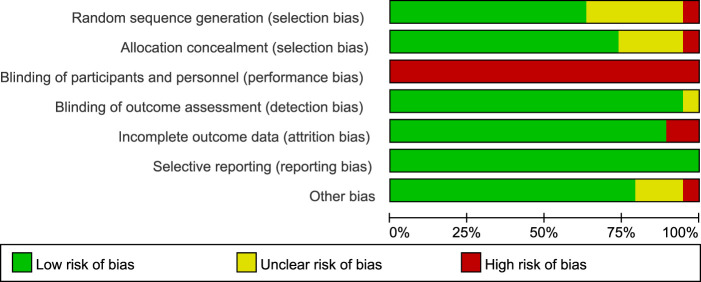
Risk of bias plot.

**FIGURE 3:**
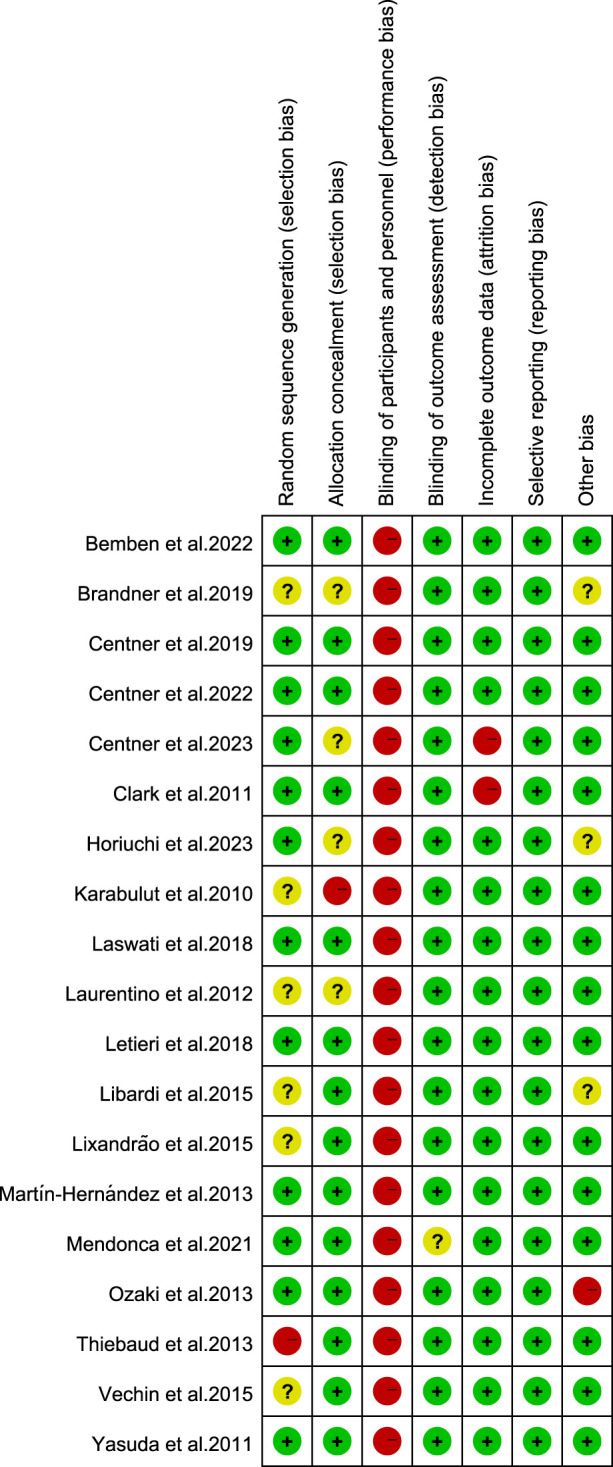
Risk of bias summary plot of included study.

The included articles were assessed using the Cochrane risk of bias tool (Figure 2; Figure 3). Regarding the selection bias, 12 articles indicated a low risk of random sequence generation, 4 articles did not report whether allocation concealment was performed, and 1 article is high risk. The blinding of subjects and researchers may not be feasible given that BFR-RT was the primary intervention in all trials; this approach resulted in a high risk of performance bias in articles. Despite reporting a high risk of performance bias, the test quality was unaffected, and 2 articles were considered to have measurement bias. Overall, no studies had reporting bias, and there were no other forms of bias in 15 articles.

### 3.4 Results of the meta-analysis

#### 3.4.1 Muscle strength adaptation: BFR-RT vs HL-RT

**FIGURE 4:**
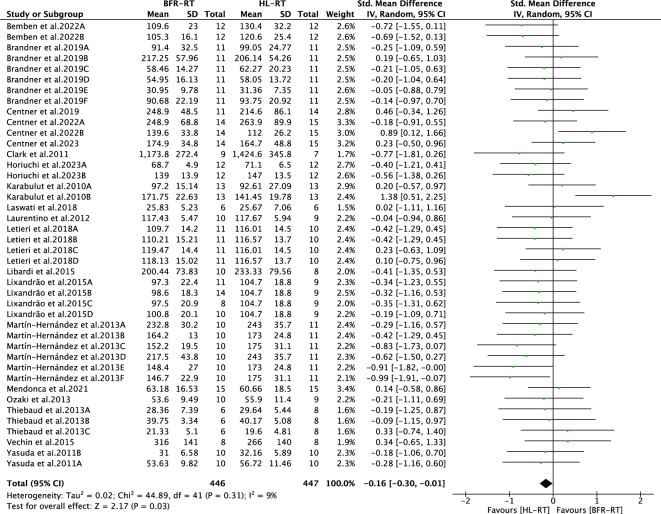
Summary results of the effect of the BFR-RT intervention on muscle strength. Different letters in the same study represent different methods of assessing muscle strength.

We included 19 articles (including 42 outcomes) for meta-analysis to compare the differences in muscle strength gain between the BFR-RT and HL-RT groups. The combined SMD showed low heterogeneity (I^2^ = 9%, *p* = 0.31). Figure 4 shows the pooled results of the meta-analysis. Statistically significant muscle strength gain was obtained in the HL-RT group compared with the BFR-RT group (SMD = −0.16, 95% CI: −0.30 to −0.01, *p* < 0.05). The heterogeneity of the muscle strength gain suggests the effect of different BFR-RT exercise protocols on muscle strength gain in healthy adults.

#### 3.4.2 Subgroup analysis of occlusion pressure prescription

**FIGURE 5:**
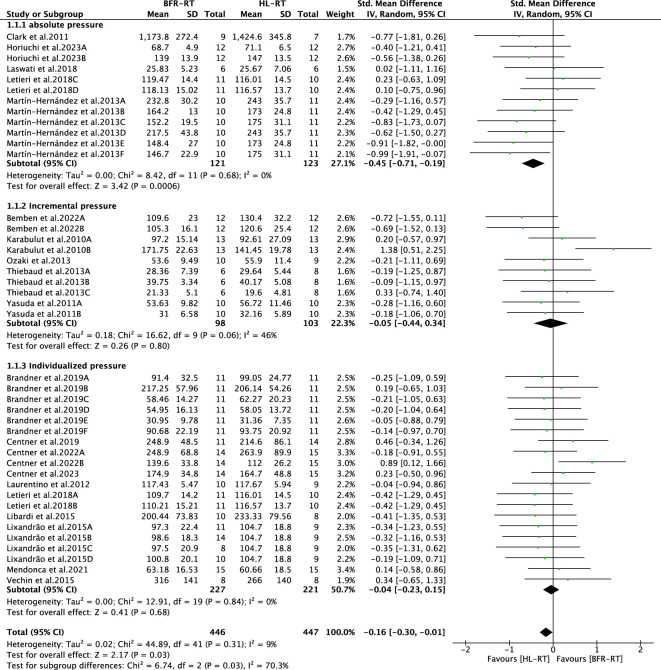
Subgroup analysis of the effect of the BFR-RT intervention on muscle strength. Different letters in the same study represent different methods of muscle strength assessment.

The results of the subgroup analysis for the BFR-RT occlusion pressure prescription (individualized, incremental, and absolute pressure) are shown in Figure 5. The muscle strength gain obtained in the HL-RT group was significantly higher than that in the BFR-RT group when absolute pressure was applied (SMD = −0.45, 95% CI = −0.71 to −0.19, *p* < 0.05, I^2^ = 0). When cuff pressure was applied in the form of incremental and individualized pressures, the muscle strength gain obtained in the HL-RT and BFR-RT groups did not show any statistically significant difference (SMD = −0.05, 95% CI = −0.44 to 0.34, *p* = 0.80, I^2^ = 46%; SMD = −0.04, 95% CI = −0.23 to 0.15, *p* = 0.68, I^2^ = 0). The subgroup tests also revealed differences between groups (*p* < 0.05), highlighting a correlation between the manner of applying cuff pressure and muscle strength gain (Figure 5).

#### 3.4.3 Subgroup analysis of cuff inflation patterns

**FIGURE 6:**
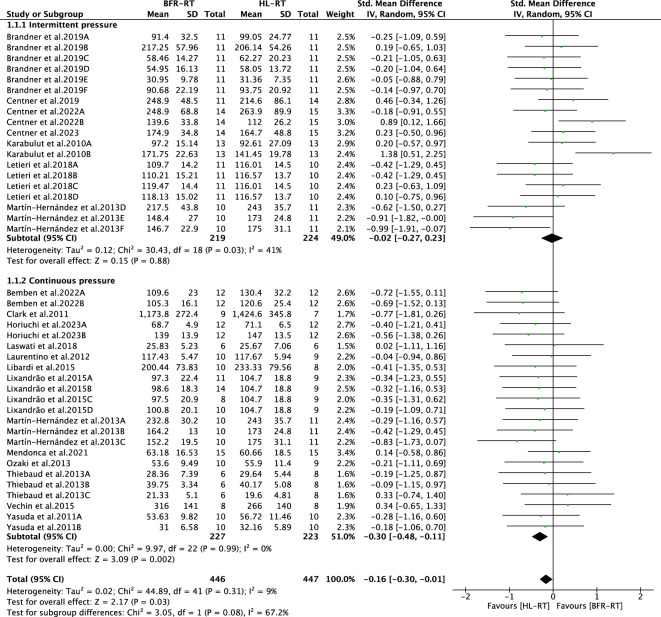
Subgroup analysis of the effect of the BFR-RT intervention on muscle strength. Different letters in the same study represent different methods of muscle strength assessment.

The results of the subgroup analysis of the BFR-RT cuff pattern of inflation (intermittent and continuous pressure) are shown in Figure 6. The difference in muscle strength gain between the BFR-RT and HL-RT groups was not significant for intermittent pressure (SMD = −0.02, 95% CI = −0.27 to 0.23, *p* = 0.88, I^2^ = 41%). Regarding the application of continuous pressure, the degree of muscle strength gain of the BFR-RT group was lower than that of the HL-RT group (SMD = −0.30, 95% CI = −0.48 to −0.11, *p* < 0.05, I^2^ = 0). The subgroup tests revealed differences between the groups (*p* < 0.05), highlighting the correlation between the cuff pattern of inflation and muscle strength gain (Figure 6).

#### 3.4.4 Subgroup analysis based on age and gender meta-regression

The findings from the meta-regression analysis revealed a significant correlation between age and changes in muscular strength adaptation (Coef. = 0.01, 95% CI = 0.001 to 0.02, *p* < 0.05), while no significant correlation was observed between gender and changes in muscular strength adaptation (Coef. = −0.17, 95% CI = −0.61 to 0.27, *p* = 0.438). Consequently, participants were classified into two groups, older and younger adults, to investigate their association with cuff pressure characteristics. Subgroup analyses demonstrated that older participants achieved comparable muscle strength adaptations to HL-RT when applied any of the exercise protocols involving cuff pressure characteristics in BFR-RT (Individualized pressures, SMD = −0.27, 95% CI = −0.72 to 0.19, *p* = 0.25, I^2^ = 0; Incremental pressures, SMD = 0.40, 95% CI = −0.02 to 0.82, *p* = 0.06, I^2^ = 47.9%; Absolute pressures, SMD = −0.17, 95% CI = −0.43 to 0.78, *p* = 0.575, I^2^ = 0; Continuous pressures, SMD = −0.02, 95% CI = −0.47 to 0.44, *p* = 0.944, I^2^ = 0; Intermittent pressures, SMD = 0.18, 95% CI = −0.16 to 0.53, *p* = 0.294, I^2^ = 57.9%); among younger participants, BFR-RT applied individualized and intermittent pressures yielded similar muscle strength adaptations as HL-RT (SMD = 0.00, 95% CI = −0.20 to 0.21, *p* = 0.963, I^2^ = 0; SMD = −0.09, 95% CI = −0.32 to 0.14, *p* = 0.462, I^2^ = 37.3%), while BFR-RT applied incremental, absolute, and continuous pressures did not produce comparable results (SMD = −0.45, 95% CI = −0.83 to −0.06, *p* < 0.05, I^2^ = 0; SMD = −0.61, 95% CI = −0.89 to −0.33, *p* < 0.05, I^2^ = 0; SMD = −0.37, 95% CI = −0.57 to −0.16, *p* < 0.05, I^2^ = 0). The findings of the meta-regression and subgroup analyses about age and gender can be observed in Supplementary Tables S3, S4.

#### 3.4.5 Combination of occlusion pressure prescription and cuff inflation patterns

**FIGURE 7:**
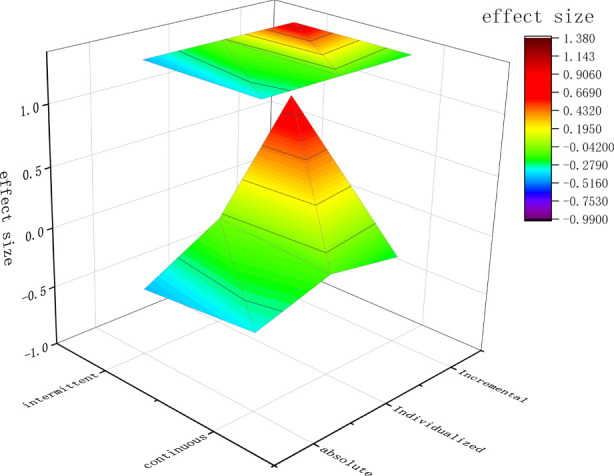
The pattern of inflation for the *X*-axis, occlusion pressure prescription for the *Y*-axis, and the effect size of the *Z*-axis.

We used 3D maps as a visual aid to analyze the cuff pressure characteristics that favor muscle strength gain. The results showed that the application of BFR-RT exercise protocols with both “individualized pressure + intermittent pressure” and “incremental pressure + intermittent pressure” produced better adaptations for muscle strength gain (Figure 7).

#### 3.4.6 Publication bias and sensitivity analysis

Finally, to assess potential publication bias in muscle strength gain, we conducted an Egger’s test and visual inspection of funnel plots, which showed that this study did not have publication bias (*p* = 0.184). In addition, sensitivity analyses were performed after individually excluding each of the 42 outcomes with respect to the effect of BFR-RT intervention on muscle strength gain. No significant changes in the combined effect values were found in the meta-analysis, indicating stable results.

## 4 Discussion

The main purpose of this meta-analysis was to compare the effects of BFR-RT on muscle strength in healthy adults. The included studies reported the effects of BFR-RT (20%–40% 1RM) on muscle strength in healthy adults and compared these with HL-RT (70%–90% 1RM) methods without BFR. The comprehensive results showed that BFR-RT produced less muscle strength gain than HL-RT, which is consistent with the results of previous studies (Lixandrao et al., 2018). Subgroup analysis of the occlusion pressure prescription showed that the BFR-RT group applying individualized pressure produced muscle strength gain comparable to the HL-RT group. Furthermore, the BFR-RT group applying incremental pressure produced muscle strength gain similar to HL-RT, whereas the BFR-RT group applying absolute pressure achieved less muscle strength gain than the HL-RT group. Subgroup analysis of the cuff pattern of inflation showed that the BFR-RT group with intermittent pressure achieved muscle strength gain comparable to that of the HL-RT group, whereas the BFR-RT group with continuous pressure achieved less muscle strength gain than the HL-RT group. Our findings also suggest that better muscle strength gain could be achieved by both “individualized pressure + intermittent pressure” and “incremental pressure + intermittent pressure” in the BFR-RT exercise protocol.

Muscle strength forms the basis for all activities in the human body. Some studies have highlighted the importance of muscle strength for specific motor skills and reduced injury rates (Suchomel et al., 2016). In the elderly population, higher muscle strength in the lower limbs is positively associated with cognitive function (Frith and Loprinzi, 2018), helps to prevent falls, and can lead to increased levels of physical activity (Trombetti et al., 2016). In the adult population, muscle strength and risk factors are negatively associated in terms of metabolic traits, higher muscle strength can reduce this relevance (Fraser et al., 2016). For special populations, muscle strength is a key factor in promoting mobility, cardiovascular capacity, and performance in daily activities (Obrusnikova et al., 2022). Researchers previously thought that the best approach for improving muscle strength is to perform HL-RT; however, as time progressed, increasing evidence showed that BFR-RT can also effectively improve muscle strength (Takarada et al., 2000; Brandner et al., 2019; Chang et al., 2022). Interestingly, many studies have focused their research objectives on comparing the differences in muscle strength gain via BFR-RT and the without vascular occlusion resistance exercise, but the effects of some characteristics of BFR-RT itself on muscle strength gain are generally ignored.

Cuff pressure characteristics are a key factor in establishing a BFR-RT exercise protocol. The choice of prescription regarding cuff occlusion pressure has also evolved, and the initial approach was to implement BFR-RT cuff pressures over arbitrary absolute pressure (Takarada et al., 2000). With the evolution of BFR-RT, the concept of initial absolute pressure became less popular. Some scholars eventually chose to gradually increase pressure during training (Karabulut et al., 2010; Bemben et al., 2022), whereas other researchers chose individualized cuff pressure based on arterial occlusion pressure (Laurentino et al., 2012; Centner et al., 2023). The rationale for the choice of incremental pressure is multifaceted, with some scholars proposing that incremental cuff pressure increases the level of perceived effort in participants (Yasuda et al., 2014b; Yasuda et al., 2015); meanwhile, others proposed that incremental pressure can help participants to better adapt to the occlusion stimulus during the initial phase of training (Junior et al., 2019a; Junior et al., 2019b). The rationale for selecting individualized pressure is that individualized pressure motion prescriptions are more accurate than broad occlusion pressure motion prescriptions when applying cuff pressure during BFR-RT exercise, with individualized prescriptions accounting for device variations (e.g., different cuff widths) when setting the same absolute pressure, thus addressing the limitations caused by this variability (Mouser et al., 2018). Overall, BFR-RT interventions using different cuff pressure characteristics affected perceived effort and adaptation to occlusive stimuli but failed to demonstrate a positive effect on muscle strength adaptation.

This meta-analysis analyzed the effect of cuff pressure characteristics on muscle strength gain. For the first time, cuff occlusion pressure prescriptions were divided into individualized, incremental, and absolute pressures for subgroup analysis. The application of individualized pressure in BFR-RT leads to muscle strength gain that is comparable to that in HL-RT. This result differs from the meta-analysis of Lixandrao et al., 2018; the heterogeneity observed in studies may be attributed to the number of included articles. In this meta-analysis, the individualized pressure in the included articles ranged between 40% and 80% limb occlusion pressure. Lixandrão et al., 2015 implemented personalized occlusion pressures of 40% and 80%, along with exercise loads of 20% and 40% of 1RM, the results of this study indicated that higher occlusion pressures yielded greater benefits for muscle strength when exercise loads were lower, whereas exercise load played a more significant role when higher exercise load were implemented. Letieri et al., 2018 implemented a personalized occlusion pressure of 80% and an exercise load ranging from 20% to 30% of 1RM over a span of 16 weeks. The participants completed a total of 48 training sessions during this period, notably, during the initial 2 weeks of the study, three sets of each exercise were executed (30, 15, 15), while in subsequent sessions, four sets were performed (15, 15, 15, 15), this discrepancy in the number of sets may lead to a more rigorous exercise stimulus at a lower pre-exercise load. In addition, in the included articles applying 50% arterial occlusion pressure, the BFR-RT intervention yielded similar muscle strength adaptations as HL-RT. Regarding incremental and absolute pressures, a recent review examined the effect of BFR-RT cuff pressure characteristics on cardiovascular metrics (Cerqueira et al., 2021). Although the significance of cardiovascular metrics in BFR-RT interventions is important, no conclusions were given regarding whether incremental or absolute pressure could be better applied in BFR-RT. In the current study, we approached this research gap from the perspective of muscle strength gain. Of the articles included in this review, five articles applied incremental pressures with pressure increases of 40, 60, and 80 mmHg, with cuff pressures ranging from 80 mm Hg to 240 mmHg, with a mean pressure of approximately 158 mmHg, and reported cuff widths ranging from 3 to 5 cm; and five articles applied absolute pressures with cuff pressures ranging from 50 mm Hg to 187.5 mmHg, with reported cuff widths ranging from 6 to 14 cm. The results of this meta-analysis showed that the BFR-RT group applied with incremental pressure showed comparable muscle strength gain to the HL-RT group. The finding differed for the BFR-RT group applied with absolute pressure.

Moreover, subgroup analysis of the cuff pattern of inflation showed that the muscle strength gain of the BFR-RT group applied with intermittent pressure was comparable to that of the HL-RT group. By contrast, the muscle strength gain of the BFR-RT group applied with continuous pressure was less apparent than that of the HL-RT group. We explained the reasons for the differences in outcomes between subgroups in the following three ways. Regarding the cause of fatigue, BFR-RT may produce higher levels of discomfort and a higher rate of perceived exertion than HL-RT without blood flow occlusion; the subjects may have manifested significant intolerance to the BFR-RT exercise regimen (Bell et al., 2018; Soligon et al., 2018; Dankel et al., 2019). The rate of perceived exertion after exercise was lower in the BFR-RT group applied with intermittent pressure; this phenomenon was not observed in the BFR-RT group applied with continuous pressure (Corvino et al., 2017). This finding may be explained by the BFR-RT applied with continuous pressure leading to an immediate onset of physiological and metabolic stress, causing increased fatigue and decreased exercise performance (Wernbom et al., 2009; Loenneke et al., 2012), suggesting greater discomfort (Neto et al., 2018; Wilk et al., 2018; Okita et al., 2019) compared with the BFR-RT group applied with intermittent pressure. From an energy supply perspective, the phosphocreatine concentration gradually decreases in BFR-RT applied with continuous pressure because of blood flow occlusion; by contrast, the release of vascular occlusion and subsequent restoration of phosphocreatine concentration through the phosphocreatine shuttle system occurs in BFR-RT applied with intermittent pressure (Suga et al., 2012; Guimarães-Ferreira, 2014; Okita et al., 2019), prompting replenishment of stored adenosine triphosphate and an increase in muscle tissue energy status (Guimarães-Ferreira, 2014). From a body metabolism perspective, BFR-RT applied with intermittent pressure provides better perfusion, venous return, and shorter occlusion times compared with BFR-RT applied with continuous pressure, with the former further preventing anaerobic respiratory byproducts from pooling and accumulating in the body, further resulting in lower levels of expected metabolic disturbances (Pearson and Hussain, 2015; Teixeira et al., 2018; Okita et al., 2019).

Furthermore, when age was considered, we found that in the elderly population, all BFR-RT exercise protocols applying different cuff pressure characteristics achieved similar muscle strength gains to HL-RT; whereas in the younger population, only two exercise protocols applying intermittent and individualized pressures achieved similar muscle strength gains to HL-RT. However, the number of included articles is small and conclusions should be drawn with caution. To provide clinical staff with more accurate BFR-RT exercise protocols, we used 3D models as an aid in investigating the combined effects of occlusion pressure prescription and cuff pattern of inflation on muscle strength gain. The results showed that the two BFR-RT exercise protocols “individualized pressure + intermittent pressure” and “incremental pressure + intermittent pressure” produced better adaptations for muscle strength gain. This finding illustrates the need to consider cuff pressure characteristics when setting up BFR-RT exercise prescriptions for healthy adults.

While focusing on the many benefits of BFR training, it is also important to consider the safety and adherence rates of the subjects.BFR training has some inherent safety concerns, as it essentially pressurizes the limb, resulting in decreased arterial blood inflow and venous blood pooling, This can result in the occurrence of ischemia and hypoxia within the body (Yasuda et al., 2010), while the inadequate elimination of lactic acid and other metabolites generated during this process is observed (Yasuda et al., 2014a; Teixeira et al., 2018). Furthermore, it has been shown that 23 min of BFR-RT training under sustained stress conditions, even with moderate exercise, can lead to muscle damage (Wernbom et al., 2020; Wernbom et al., 2021). However, Nakajima et al.'s questionnaire survey of 12,600 individuals who had experienced BFR training found that the most common side effects of BFR were subcutaneous hemorrhage and temporary numbness, but that this response diminished and disappeared as the cuff was released and training progressed (Nakajima et al., 2006). Therefore, from the point of view of safety and compliance, the BFR-RT with intermittent compression and the BFR with incremental pressure protocols may be a good choice for physical training. This is because with intermittent pressurization, subjects can release the cuff pressure restriction during rest breaks between sets, and electronic compression devices can be easily deflated and inflated during rest breaks without interfering with the training process; intermittent pressurization is also a viable option for people with low tolerance for ischemic pain and discomfort, and can improve adherence ineffective this population (Davids et al., 2021); and incremental pressurization can allow the subject to have a process of adapting to the pressure during training (i.e., cuff pressure can be slowly increased), which would be safer for participants and have a more positive attitude toward subsequent training. Coupled with the fact that our findings also suggest that intermittent pressurized BFR and incremental pressurized BFR exercise interventions have positive muscle strength growth adaptations, we recommend the use of intermittent pressurized incremental pressurized BFR-RT exercise regimens for long-term BFR-RT training, which appears to improve subject safety and compliance rates as well as better training outcomes.

## 5 Limitations

First, the vast majority of the population included in this study was inexperienced in training, which provides limited guidance for those undergoing resistance training. Second, there is a lack of research on young female experimenters, and future research could focus on this population. Thirdly, the duration of the training period spanned a considerable timeframe ranging from 4 to 16 weeks, potentially influencing the observed results. Finally, considering multiple outcomes from the same study in a meta-analysis may also partially affect the homogeneity of results.

## 6 Conclusion

Our findings indicate that, in general, HL-RT produces greater muscle strength gains than BF-RT. However, upon further analysis, we observed that BFR-RT can yield similar muscle strength gains to HL-RT when considering specific cuff pressure characteristics, such as individualized pressure, incremental pressure, and intermittent pressure BFR-RT exercise protocols. Additionally, our results indicate that the combination of “individualized pressure + intermittent pressure” and “incremental pressure + intermittent pressure” BFR-RT exercise protocols display a tendency towards enhanced muscle strength gains. Overall, cuff pressure characteristics should be given more attention when setting up BFR-RT exercise protocols. It is important to note that age affects subgroup outcomes, indicating that the age factor should also be considered when considering the cuff pressure characteristics of the BFR-RT.

## Data Availability

The original contributions presented in the study are included in the article/Supplementary Material, further inquiries can be directed to the corresponding author.
