# Supplementary material for: Muscle strength adaptation between high-load resistance training versus low-load blood flow restriction training with different cuff pressure characteristics: a systematic review and meta-analysis
Source: Front Physiol. 2023 Aug 25;14:1244292. doi: 10.3389/fphys.2023.1244292 (PMC10485702; doi:10.3389/fphys.2023.1244292)
Supplement: Supplementary file 1 [file DataSheet1.docx]

Supplementary Material

**Effect of different cuff pressure blood flow restriction combined with resistance training on muscle strength in healthy adults: A systematic review and meta-analysis**

Hualong Chang, Jing Yan, Guiwei Lu, Biao Chen, Jianli Zhang*

*** Correspondence:** Jianli Zhang: zhangjl@zjnu.edu.cn

# Supplementary Figures and Tables

## Supplementary Figures

**
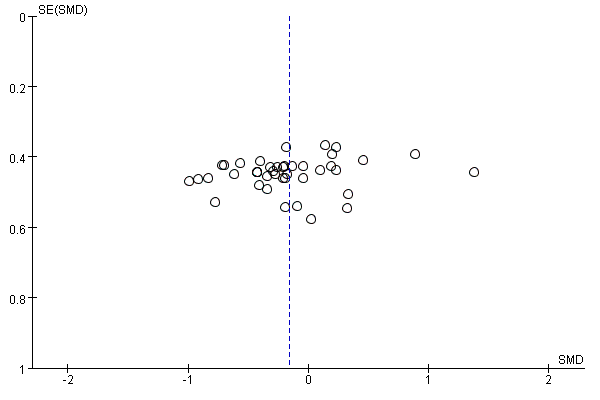
**

**Supplementary Figure S1.** Funnel plot of the effect of BFR-RT cuff pressure characteristics on muscle strength.

## Supplementary Tables

**Supplementary Table S1**. Search strategy to identify the relevant articles on PubMed.

| **PubMed:** | ("Blood Flow Restriction Therapy"[Mesh] OR BFR Therapy[Title/Abstract] OR BFR Therapies[Title/Abstract] OR Therapy, BFR[Title/Abstract] OR Blood Flow Restriction[Title/Abstract] OR KAATSU[Title/Abstract] OR BFRT[Title/Abstract] OR restricted leg blood flow[Title/Abstract] OR restricted leg muscle blood flow[Title/Abstract] OR blood flow occlusion[Title/Abstract] OR blood flow restricted clastic band training[Title/Abstract] OR Occlusion training[Title/Abstract] OR occluded blood flow[Title/Abstract] OR restricted blood flow[Title/Abstract] OR vascular restriction[Title/Abstract] OR vascular occlusion[Title/Abstract]) AND ("Muscle Strength"[Mesh] OR Strength, Muscle[Title/Abstract] OR Arthrogenic Muscle Inhibition[Title/Abstract] OR Arthrogenic Muscle Inhibitions[Title/Abstract] OR Inhibition, Arthrogenic Muscle[Title/Abstract] OR Muscle Inhibition, Arthrogenic[Title/Abstract] OR repetition maximum[Title/Abstract] OR 1RM[Title/Abstract] OR MVC[Title/Abstract] OR maximum voluntary contraction[Title/Abstract] OR MVIC[Title/Abstract] OR maximal voluntary isometric contraction[Title/Abstract] OR muscle force[Title/Abstract] OR skeletal muscle[Title/Abstract] OR muscle fiber[Title/Abstract] OR muscle power[Title/Abstract] OR isokinetic[Title/Abstract] OR isometric[Title/Abstract] OR muscle force[Title/Abstract] OR strength[Title/Abstract] OR dynamic[Title/Abstract]) AND (randomized controlled trial[Publication Type] OR randomized[Title/Abstract] OR placebo[Title/Abstract] OR RCT[Title/Abstract] OR random*[Title/Abstract] OR triple blind*[Title/Abstract] OR clinical trial[Title/Abstract] OR allocation[Title/Abstract] OR single blind[Title/Abstract] OR double blind[Title/Abstract]) |
| --- | --- |

We have followed the above search strategy to find the relevant articles from the PubMed database.

**Supplementary Table S2.** Subgroup evaluation criteria

| Occlusion pressure prescriptions | Individualized pressures | | Pressure setting based on limb occlusion pressure or arterial occlusion pressure |
| --- | --- | --- | --- |
|  | non-Individualized | Incremental pressures | Elevated cuff pressure throughout the duration |
|  |  | Absolute pressures | Cuff pressure remains fixed throughout the duration |
| Cuff inflation patterns | Intermittent pressures | | Removing cuff pressure during exercise intervals |
|  | Continuous pressures | | Maintaining cuff pressure during exercise intervals |

**Supplementary Table S3.** Meta-regression analysis of Age and Gender.

| Experimental Intervention | Coefficient | Standard Error | T-Value | p-Value | [95% Conf. Interval] | |
| --- | --- | --- | --- | --- | --- | --- |
| Gender | –.1703339 | .217343 | –0.78 | 0.438 | –.6099515 | .2692838 |
| Age | .0093697 | .0042414 | 2.21 | 0.033 | .0007976 | .0179419 |

**Supplementary Table S4.** Subgroup analysis of the effect of the BFR-RT cuff pressure characteristics on muscle strength in different age groups.

| Variable | Young adults | | | | Old adults | | | |
| --- | --- | --- | --- | --- | --- | --- | --- | --- |
|  | No. Of trials | SMD (95%CI) | I^2^% | P | No. Of trials | SMD (95%CI) | I^2^% | P |
| Individualized pressures | 7 | 0.00(–0.20 to 0.21) | 0 | 0.963 | 3 | –0.27(–0.72 to 0.19) | 0 | 0.250 |
| Incremental pressures | 3 | –0.45(–0.83 to –0.06) | 0 | 0.023 | 2 | 0.40(–0.02 to 0.82) | 47.9 | 0.060 |
| Absolute  pressures | 4 | –0.61(-0.89 to –0.33) | 0 | 0.000 | 1 | –0.17(–0.43 to 0.78) | 0 | 0.575 |
| Continuous pressures | 10 | –0.37(–0.57 to –0.16) | 0 | 0.000 | 3 | –0.02(–0.47 to 0.44) | 0 | 0.944 |
| Intermittent pressures | 5 | –0.09(–0.32 to 0.14) | 37.3 | 0.462 | 2 | 0.18(–0.16 to 0.53) | 57.9 | 0.294 |

**Supplementary Table S5.** Characteristics of the included studies.

| Study | Exercise  Mode | Frequency  (t/wk) | Cuff Pressure  (mm Hg) | Cuff Width  (cm) | occlusion pressure prescriptions | cuff inflation patterns |
| --- | --- | --- | --- | --- | --- | --- |
| Bemben et al., 2022 | Knee extension  Knee flexion | 3 | Range, 160-200  Mean, 180 | 5 | Incremental | Continuous |
| Brandner et al., 2019 | Knee extension  Back squat  Calf raises  Bench press  Seated row  Biceps curl | 3 | 60% LOP  Lower-Limb, 108  Upper-Limb, 80.5 | 10.5 | Individualized | Intermittent |
| Centner et al., 2019 | Standing and sitting calf-raises | 3 | 50% AOP  120 | 12 | Individualized | Intermittent |
| Centner et al.,2022 | Leg press  Knee extensions  Standing and sitting calf-raises | 3 | 50% AOP  120 | 12 | Individualized | Intermittent |
| Centner et al., 2023 | Standing and sitting calf-raises  Knee extensions,  Leg press  Lat pull  Bench press | 3 | 50% AOP  120 | 12 | Individualized | Intermittent |
| Clark et al., 2011 | Knee extension | 3 | 1.3 times of individual systolic blood pressure | 6 | Absolute | Continuous |
| Horiuchi et al., 2023 | Knee Extensions  Leg presses | 4 | 1.3 times of individual systolic blood pressure | 11 | Absolute | Continuous |
| Laswati et al.,2018 | Biceps curl | 2 | 50 | 13 | Absolute | Continuous |
| **Supplementary Table S5.** Cont. | | | | | | |
| Study | Exercise  Mode | Frequency  (t/wk) | Cuff Pressure  (mm Hg) | Cuff Width  (cm) | occlusion pressure prescriptions | cuff inflation patterns |
| Karabulut et al., 2010 | Leg press  Leg extension | 3 | Range, 160-240  Mean, 205.5 | NR | Incremental | Intermittent |
| Laurentino et al., 2012 | Knee extension | 2 | 80% AOP  94.8 | 17.5 | Individualized | Continuous |
| Libardi et al., 2015 | Leg press | 2 | 50%AOP  67 | 17.5 | Individualized | Continuous |
| Letieri et al., 2018 | Squat  Leg Press  Knee Extension  Leg Curl | 3 | 80% A0P  105.45/185.75 | NR | Absolute/Individualized | Intermittent |
| Lixandrão et al., 2015 | Knee extension | 2 | 40% AOP/80% AOP  55.5/109.6/54.5/105.0 | 17.5 | Individualized | Continuous |
| Martín-Hernández et al., 2013 | Knee extension | 2 | 110 | 14 | Absolute | Intermittent/Continuous |
| Mendonca et ai., 2021 | Plantar-flexion | 5 | 60% AOP | 13 | Individualized | Continuous |
| Ozaki et al., 2013 | Bench press | 3 | Range, 100-160  Mean, 148.33 | 3 | Incremental | Continuous |
| Thiebaud et al., 2013 | Seated chest press  Seated row  Seated shoulder press | 3 | Range, 80-120  Mean, 108.13 | 3.3 | Incremental | Continuous |
| Vechin et al., 2015 | Leg press | 2 | 50% AOP  71 | 18 | Individualized | Continuous |
| Yasuda et al., 2011 | Bench press | 3 | Range, 100-160  Mean, 148.33 | NR | Incremental | Continuous |

t, times; wk, weeks; LOP, Limb Occlusion Pressure; AOP, Arterial Occlusion Pressure；NR, unreported
